# The impact of BMI on psychological health in oldest old individuals–Are there differences between women and men?

**DOI:** 10.1371/journal.pone.0283089

**Published:** 2023-03-29

**Authors:** Franziska U. C. E. Jung, Sina Gerhards, Melanie Luppa, Margrit Löbner, Steffi G. Riedel-Heller

**Affiliations:** Institute of Social Medicine, Occupational Health and Public Health, Faculty of Medicine, Leipzig University, Leipzig, D-Germany; Shahrood University of Medical Sciences, ISLAMIC REPUBLIC OF IRAN

## Abstract

**Objective:**

The aim of this study was to determine the association of mental health issues associated with BMI and gender in the oldest old population (secondary data analyses).

**Method:**

The data were taken from the second follow-up of a long-term study investigating the impact of the COVID-19 pandemic on health in oldest old individuals (range: 77–96 years). The response rate was 80.0%. Apart from sociodemographic characteristics (age, gender, weight and height); anxiety, depression, somatic complaints and social support were assessed in this survey.

**Results:**

Analyses revealed gender-specific differences, indicating that male participants with excess weight show more complaints compared to their counterparts without excess weight. According to regression results, BMI was associated with somatization, but not depression or anxiety.

**Conclusion:**

High BMI contributed to more somatic complaints and men may be affected differently by BMI regarding their mental well-being. Longitudinal results are needed in order to confirm these findings and develop suitable interventions based on individual needs of the oldest old.

## Introduction

Overweight and obesity are currently seen as major public health concern worldwide, characterized by a rising prevalence in men and women.

In general, studies find lower prevalences of obesity in the oldest old (27.8%) compared to adults aged between 65 and 74 years (40.8%) [1]. This was confirmed by a longitudinal study in a sample of individuals aged 85 years and older, where the probability of obesity decreased with age [2]. However, the authors also find that almost half of the participants had excess weight, suggesting that this may be a major challenge for public health. In fact, being on the extreme ends of the Body Mass Index (BMI) spectrum has been shown to be associated with an increased risk for mortality in older individuals due to the shared co-morbidity between age and obesity, such as coronary heart disease, diabetes and hypertension. Therefore, in tandem with an increasing age of the general population, it is also important to investigate prevalence and consequences of extreme weight gain in the oldest old (i.e. adults aged 75 and older), in order to prepare the health care system in terms of appropriate care and associated costs.

In general, individuals with overweight and obesity have been shown to suffer from several health impairments, such as coronary heart disease, Type 2 diabetes, hypertension and mental health issues such as depression and anxiety [3, 4]. Interestingly, in older people, overweight and obesity have been rather identified as possible protective factors in cardiovascular outcomes, also known as the obesity paradox [5, 6]. In addition, the obesity-mental health association seems to be more dominant in late life than early or middle adulthood [7]. Currently, not much is known about the link between overweight and obesity and mental health in the oldest old, especially with regard to possible gender-differences that have been found in other age cohorts. People aged 75 and older are the fastest growing population group. Due to the demographic development, the topic is the topic under investigation is particularly important.

Therefore, the aim of this secondary data analysis was to shed light on possible effects of BMI on psychological well-being in a sample of late elderly people.

## Method

The data were taken from the second follow-up of a long-term study investigating the impact of the COVID-19 pandemic on overall health, mental health as well as stress and coping in oldest old individuals (range: 77–96 years). In this observational study, 378 community-dwelling senior persons, living in Leipzig (Germany) or nearby, were contacted and offered to participate. The assessment was conducted during the first wave of the COVID-19 pandemic (March to July 2020). The sample was chosen from the institute’s database of possible participants who had previously participated in population- and primary care-based old age studies and had given their consent to be approached for upcoming research.

For the purpose of the current study, 170 Participants were contacted at the the end of 2021 and the beginning of 2022 for the second follow-up wave. In this study wave, the response rate was 80.0%. Due to missing data on height and weight, 15 cases had to be excluded and a remaining sample of n = 121 was used for analysis.

The study has been approved by the Ethics Committee of the Medical Faculty of the University of Leipzig (approval number: 206-20-ek). Written consent on data use for analysis was obtained from all participants. More details on the method, instruments and procedure of the overall observational study (including details on data collection and purpose of the study) can be found in a previously published study [8].

## Instruments

Apart from sociodemographic information (e.g. age, gender, marital status), questions on social support using the 5-item German Version of the ENRICHD Social Support Inventory (ESSI-D, Cronbach alpha: 0.87, [9]), as well as the Brief Symptom Inventory (BSI-18, [10]), including three subscales on depression (Cronbach alpha: 0.70) and anxiety (Cronbach alpha: 0.65) were included. In order to assess general health, items from the subscale on somatic complaints were also considered (Cronbach alpha: 0.68). The items of the BSI-18 can be answered using a five-point Likert scale (0 = not at all, 4 = very strongly). The scale has been shown to have good psychometric properties [11]. The ESSI-D, which includeds five items and a five-point-Likert scale (1 = never; 5 = always) is an established instrument with good psychometric properties [9, 12]. In addition, the visual analogue scale EQ-VAS was used to assess the current overall health status [13]. The EQ-VAS includes one item asking for the current overall health status. Answers can range from 0 (= worst health status) to 100 (= best health status).

The Body Mass Index (BMI) was calculated in accordance with the formular and categories provided by the World Health Organization (BMI = kg/m^2^, [14]).

Education was determined using the CASMIN classification (low, middle, high, [15]).

Covid-19 status was assessed based on self report. Participants were asked the following question: “Do you know anyone who has been diagnosed with COVID-19?”. Individuals were categorized as COVID-19 positive if they agreed with the statement “I have been diagnosed with COVID-19 myself” and negative if they disagreed with this statement. This categorization may not reflect their COVID-19 status at the time of the data collection.

### Statistical analyses

Data were analyzed descriptively, using Chi^2^-square (x^2^) tests and Wilcoxon rank sum tests in order to determine group differences (related to either gender or BMI-category). In addition, multivariate regression analyses were conducted. Due to the distribution of the outcome variable, generalized linear regression models were calculated (including gamma distribution and log link function, [16]). For the purpose of regression analysis, BMI was dichotomized into two categories: underweight and normal weight if BMI was lower or equal 24.9, and overweight and obese if BMI was equal or greater 25.0.

The statistical software program Stata 16.0 SE (College Station, Texas, USA) has been used for all analyses.

## Results

Descriptive information is summarized in Table 1. Overall, 55.4% were female and 44.6% were male. The mean age of the overall sample was 86.7 years. Regarding the BMI, 2.5% were underweight, 47.9% were normalweight, 33.9% had overweight and 15.7% of the overall sample had obesity. Regarding COVID-19, only 3.3% (n = 4) of the overall sample have ever been diagnosed with COVID-19. Therefore, we did not inlcuded this variable into regression analyses.

**Table 1:** Descriptive information on the overall sample and the two sub-samples with regard to gender.

	Overall sample	Men (n = 54)	Women (n = 67)	p-value
	(n = 121)			
Age^1^	Ø 86.7 (SD: 4.4)	Ø 87.5 (SD: 4.5)	Ø 86.1 (SD: 4.3)	n.s.
Body Mass Index (BMI)^2^	Ø 26.0 (SD: 3.9)	Ø 26.0 (SD: 3.9)	Ø 25.4 (SD: 4.4)	n.s.
≤24.9	61 (50.4%)	26 (48.1%)	35 (52.2%)	
≥25.0	60 (49.6%)	28 (51.8%)	32 (47.8%)	
Marital Status				p < 0.001
married	53 (44.2%)	35 (64.8%)	18 (27.7%)	
Single	12 (10.0%)	4 (7.4%)	8 (12.3%)	
divorced	54 (45.0%)	15 (27.8%)	39 (60.0%)	
Education (CASIM)				n.s.
low	39 (32.5%)	15 (27.8%)	24 (36.4%)	
Middle	32 (26.7%)	12 (22.2%)	20 (30.3%)	
high	49 (40.8%)	27 (50.0%)	22 (33.3%)	
Overall Health (VAS)^3^, n = 118	Ø 60.0 (SD: 18.8)	59.1 (SD: 20.2)	60.8 (SD: 17.8)	n.s.
ESSI^4^, n = 116	Ø 22.1 (SD: 3.6)	Ø 22.4 (SD: 3.7)	Ø 21.8 (SD: 3.4)	n.s.
Depression^5^	Ø 2.3 (SD: 3.1)	Ø 2.2 (SD: 3.5)	Ø 2.3 (SD: 2.9)	n.s.
Anxiety^6^	Ø 2.3 (SD: 2.7)	Ø 1.9 (SD: 2.3)	Ø 2.6 (SD: 2.9)	n.s.
Somatization^7^	Ø 3.9 (SD: 3.9)	Ø 3.7 (SD: 3.3)	Ø 4.1 (SD: 2.9)	n.s.

Note

^1^ = range: 77–96 years

^2^ = according to WHO [14]

^3^ = range: 10–90

^4^ = range: 4–20

^5^n = 118

^6^n = 117

^7^n = 115

p-values with regard to group differences were obtained from chi square tests (categorical variables) and Wilcoxon rank sum tests (continuous variables)

Furthermore, female and male participants did not show any significant difference with regard to age, or education. In addition, no significant differences between women and men were found regarding BMI, BMI category (underweight, normal weight, overweight, obesity) or excess weight (BMI<25 vs. BMI<25). However, with regard to marital status differences were observed, as men were more likely to be married and women were more likely to be divorced (p<0.001). Concerning the independent variables under investigation, men did not differ from women in terms of overall health status, depression, anxiety, somatization or social support.

**Fig 1:**
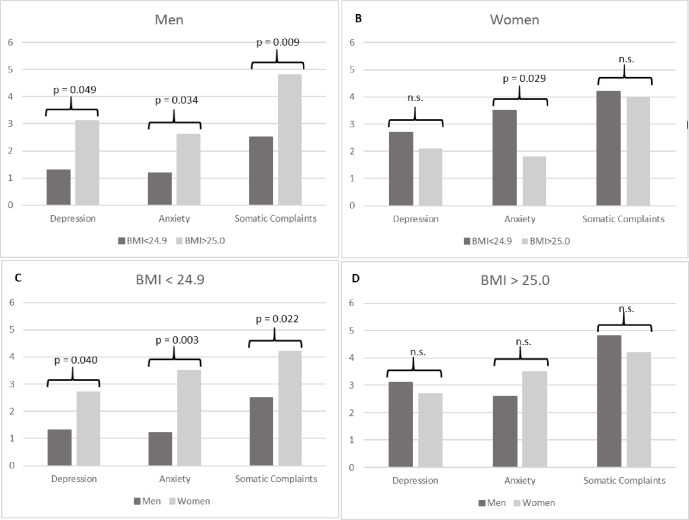
Symptoms of depression, anxiety and somatic complaints in relation to gender and body mass index. Note: A–male participants with or without excess weight; B–female participants with or without excess weight; C–participants without excess weight (women vs. men); D–participants with excess weight (women vs. men).

Furthermore, symptoms of depression and anxiety as well as somatic complaints are summarized in Fig 1. Within the male sub-sample, individuals with excess weight (BMI >25.0) show significantly more symptoms compared to individuals with a BMI lower than 25. Within the female sub-sample, significant differences were only obtained with regard to symptoms of anxiety, as women without excess weight exhibited greater symptom severity compared to their counterparts with excess weight.According to the results, significant differences between men and women were only found in individuals without excess weight, as greater symptom severity across all three outcomes were observed in women.

BMI-category (underweight/normal weight vs. overweight/obese) did not affect overall health, social support, independent of gender (see Table 1 and S1 Appendix). However, men who reported a BMI greater than 25 exhibited higher scores on the scales measuring depression, anxiety and somatization. Interestingly, significant results for women could only be obtained with regard to anxiety, where being underweight or normal weight was associated with greater anxiety.

In addition, results of the generalized linear regression models is summarized in Table 2. Here, BMI was only significantly related to somatization, but not depression or anxiety, when controlling for sociodemographic factors, social support and overall health.

**Table 2:** Generalized logistic regression models with three dimensions of psychological health as outcome variables.

	Model 1: Somatization (obs.: 103)		Model 2: Depression (obs.: 106)		Model 3: Anxiety (obs.: 105)	
	coef	95% CI	coef	95% CI	coef	95% CI
BMI (ref: BMI<24.9)^1^	**0.384**	0.099–0.670	0.584	-0.012–1.181	0.129	-0.403–0.661
Age	**0.033**	0.003–0.064	0.016	-0.054–0.085	0.036	-0.037–0.109
Gender (ref: female)	**-0.368**	-0.691–0.044	0.106	-0.499–0.712	-0.530	-1.121–0.061
Marital Status						
single	0.136	-0.200–0.472	0.013	-0.532–0.558	-0.256	-0.867–0.354
divorced	0.022	-0.517–0.561	0.350	-0.445–1.136	-0.024	-0.498–0.545
Education (CASIM)						
middle	0.054	-0.274–0.382	0.107	-0.531–0.745	0.155	-0.380–0.690
high	0.150	-0.220–0.519	0.028	-0.610–0.666	0.056	-0.550–0.661
Overall Health (VAS)	**-0.020**	-0.027 –-0.013	-0.015	-0.030 –-0.001	**-0.013**	-0.025 –-0.001
Social Support (ESSI)	0.006	-0.042–0.054	-**0.113**	-0.175 –-0.050	-0.023	-0.088–0.042

Note: obs. = number of observations in this model; BMI was dichotomized into 0 = BMI <24.9 and 1 = BMI >25.0

## Discussion

The aim of this short report was to give first insights into possible associations between BMI and psychological health in the oldest old population. Even if prevalences of obesity in the oldest old are lower compared to other age groups [2], it has been shown that this rise is currently much more pronounced, especially in individuals aged 85 to 89 [17]. Regarding the current weight status, almost half of the individuals in the current sample were overweight or obese, replicating results of a similar study [2]. Obesity and overweight have often been linked to mental health problems such as symptoms of depression or anxiety. Research has shown that mental health symptoms change over time, especially when individuals transit from old to oldest old [18]. At the same time, more depressive symptoms are significantly associated with multimorbiditiy among oldest-old individuals [19]. Concerning the study aim, somatization was the only outcome that appeared to be affected by BMI. In other words, being overweight or obese was associated with greater somatization. No such relation was found regarding depressiveness or anxiety. The latter has been suggested previously, analyzing a sample of individuals aged 85 years and older, where the probability of obesity was not associated with depression [2]. Moreover, descriptive analyses in our study suggest, that men may be more negatively affected by their BMI. Compared to the subsample with normal weight, male participants with overweight and obesity show significantly greater symptoms of depression, anxiety and somatization. Interestingly, female participants with a BMI below 25 show significantly more anxiety than their counterparts with a BMI of 25 and higher, indicating that the “obesity paradox” described earlier may also account for BMI-related differences in mental health [20, 21].

Previous research has underlined the importance of focussing on gender specific approaches in mental health research [22], however, it has alos been suggested that larger gender differences can rather be found for symptoms with clinical relevance (such as mayor depression) but not symptoms per se [23]. Contrary to our results, other studies generally find associations between obesity and depression in women, but not in men, due to a stronger genetic disposition to excess weight and depressed mood [24–26]. However, previous research focussed on younger age cohorts. So far, evidence explaining this association in the oldest old population is lacking. Therefore, based on the results of the current study, prospective research should investigate the underlying psychological or physiological processes that explain these gender differences in the association between BMI and psychological health.

Moreover, it is important to further investigate synergic effects of overweight and obesity on psychological well-being in the oldest old population. Future research should mirror causal relationships using longitudinal designs in order to develop suitable interventions for this vulnerable group based on individual needs, especially since both–weight as well as mental health status–change when transitioning from old to oldest-old [18]. In addition, parameters such as the onset of overweight and obesity should be included into prospective study designs (ie. whether excess weight existed over the lifespan) as this may determine the onset of depression, especially in women [26]. Geriatric assessments should be person-centered, especially in terms of associations between excess weight and mental health.

The study has some limitations, such as small sample size as well as self-reported weight and height. The data has been collected during the COVID-19 pandemic, which may have biased the results. No information on income or relevant comorbidities (for instance regarding cognitive impairments or hypertension) were obtained, which should be included in future studies as a confounding variable.

## Supporting information

S1 Checklist STROBE statement—checklist of items that should be included in reports of observational studies. (PDF)

S1 Appendix (DOCX)
