# Supplementary material for: The impact of BMI on psychological health in oldest old individuals–Are there differences between women and men?
Source: PLoS One. 2023 Mar 29;18(3):e0283089. doi: 10.1371/journal.pone.0283089 (PMC10058076; doi:10.1371/journal.pone.0283089)
Supplement: S1 Appendix — (DOCX) [file pone.0283089.s002.docx]

Appendix

Figure

|  | **Overall sample**  **(n = 121)** | **Men (n = 54)** | | | **Women (n = 67)** | | |
| --- | --- | --- | --- | --- | --- | --- | --- |
|  |  | **BMI<25** | **BMI >25** | **p-value** | **BMI<25** | **BMI >25** | **p-value** |
| BSI-18  Depression^5^  Anxiety^6^  Somatization^7^ | Ø 2.3 (SD: 3.1)  Ø 2.3 (SD: 2.7)  Ø 3.9 (SD: 3.9) | Ø 1.3 (SD: 2.1)  Ø 1.2 (SD: 1.5)  Ø 2.5 (SD: 2.5) | Ø 3.1 (SD: 4.2)  Ø 2.6 (SD: 2.6)  Ø 4.8 (SD: 3.6) | z=-1.972, p=0.049  z=-2.116, p=0.034  z=-2.611, p=0.009 | Ø 2.7 (SD: 3.3)  Ø 3.5 (SD: 3.4)  Ø 4.2 (SD: 2.9) | Ø 2.1 (SD: 2.4)  Ø 1.8 (SD: 2.1)  Ø 4.0 (SD: 3.1) | n.s.  z=2.187, p=0.029  n.s. |
